# Elevated pCO_2_ affects behavioural patterns and mechano-sensation in predatory phantom midge larvae *Chaoborus obscuripes*

**DOI:** 10.1038/s41598-020-58763-4

**Published:** 2020-02-04

**Authors:** Adrianna A. Kowalewska, Nina Krebs, Ralph Tollrian, Linda C. Weiss

**Affiliations:** 1 Ruhr- University Bochum, Department for Animal Ecology, Evolution and Biodiversity, Universitaetsstraße 150, 44780 Bochum, Germany; 2 Alfred-Wegener-Institute, Helmholtz Centre for Polar and Marine Research, Department for Integrative Ecophysiology, Am Handelshafen 12, E-1555, 27570 Bremerhaven, Germany

**Keywords:** Climate-change ecology, Ecology

## Abstract

Aquatic acidification is a major consequence of fossil fuel combustion. In marine ecosystems it was shown, that increasing pCO_2_ levels significantly affect behavioural and sensory capacities in a diversity of species. This can result in altered predator and prey interactions and thereby change community structures. Just recently also CO_2_ dependent acidification of freshwater habitats has been shown. Also here, increased levels of pCO_2_ change organisms’ behaviour and sensory capacities. For example, the freshwater crustacean *Daphnia’s* ability to detect predators and accurately develop morphological defences was significantly reduced, rendering *Daphnia* more susceptible to predation. It was speculated that this may have cascading effects on freshwater food webs. However, for a comprehensive understanding of how increased levels of CO_2_ affect trophic interactions, it is also important to study how CO_2_ affects predators. We tested this using the dipeteran phantom midge larva *Chaoborus obscuripes*, which is a world-wide abundant inhabitant of freshwater impoundments. We monitored activity parameters, predation parameters, and predation rate. *Chaoborus* larvae are affected by increased levels of pCO_2_ as we observed an increase in undirected movements and at the same time, reduced sensory abilities to detect prey items. This is likely to affect the larvae’s energy budgets. *Chaoborus* is a central component of many freshwater food-webs. Therefore, CO_2_ effects on predator and prey levels will likely have consequences for community structures.

## Introduction

The earth’s climate is currently changing at a fast rate due to the ongoing release of greenhouse gases like CO_2_ into the atmosphere. A large portion of this CO_2_ is taken up by the oceans, changing seawater chemistry and reducing pH with consequences for marine ecosystems^1,2^. Lately it was shown, that CO_2_ also accumulates in freshwater habitats, also changing pH^3–5^. This probably has been long overlooked as in freshwater environments CO_2_ conditions are highly divers^6^. Here they depend on the geographic location and respective climatic regimes, heterotrophic activity in combination with a complexity of abiotic and biotic interactions, which is further complicated by soil respiration rates, and terrestrial productivity^7^. All these factors contribute to the overall freshwater pCO_2_ which can therefore also be higher than atmospheric pCO_2_^4,8^. In fact, pCO_2_ in freshwater lakes world-wide ranges from 3.1-fold below to 16-fold above atmospheric pCO_2_, with a mean of ~1000 µatm in 2007^6,8,9^. Moreover, in freshwater systems pCO_2_ is often not stable throughout the day and throughout the season^10^. Regardless, authors have discussed^7^, prognosticated^5^ and shown^3^ that also freshwaters acidify with ongoing fossil fuel combustion. It is further discussed that pCO_2_ peak periods intensify under climate change scenarios^3,7^.

Elevation of environmental pCO_2_ levels accompanied by changes in aquatic pH has detrimental effects on organism fitness. Ocean acidification not only affects calcifying organisms where it reduces calcification abilities and growth rates^1^ but also affects development^11^, reproduction^12^, metabolic rate^13^, sensory abilities and behaviour in a range of non-calcifying species^14,15^. Especially, when sensory abilities are impeded, this can change species interactions as organisms are hampered in their ability to detect con- and heterospecifics. For example, sensory cues passing between predator and prey cannot be correctly interpreted and anti-predatory responses are often suppressed which may result in altered community dynamics. This has been displayed in a range of marine fish where pCO_2_ dependent reductions in pH affect sensory abilities. As a result, this can impair the detection of predators^16–18^. Moreover, behaviour is affected rendering some prey more active, so that prey is more vulnerable to predators^18^.

Similar observations have been made in freshwater prey species. For example, pink salmon larvae *Oncorhynchus nerka* show alterations in olfactory responses and anti-predator behaviour towards elevated pCO_2_^19^. Similarly, shelter seeking behaviour in crayfish is affected^20^. More explicitly, in the freshwater crustacean *Daphnia* (which is a keystone species as it has a disproportionally large effect on its natural environment as it links primary produces to higher trophic levels), it was shown that sensory abilities are impaired by elevated levels of pCO_2_^3^. In two species (i.e. *D. pulex* and *D*. *longicephala*) the ability to sense predators and develop accurate morphological defences was hampered, which renders them more susceptible to predation. This was discussed to have far reaching effects for the ecosystem as an inadequate defence expression may have cascading effects on all trophic levels^3^. However, the increasing prey vulnerability is just one side of pCO_2_ impacts on predator-prey systems. To our knowledge, possible effects of constantly elevated pCO_2_ levels on freshwater predators and their predation rates have not been shown. To uncover this, we here investigated the effect of increased pCO_2_ levels on one central predator preying on first level consumers. The phantom midge larvae *Chaoborus* (diptera) is a typical inhabitant of standing freshwater bodies world-wide^21^. While they serve as an important food source for higher trophic levels including many fish species, the larvae themselves prey on ciliates, copepods, and cladocerans like *Daphnia*^22,23^. If the predator is affected by elevated levels of pCO_2_, and predation effectivity is reduced the overall food web effects become less straight-forward.

## Results

### Activity patterns

**Figure 1:**
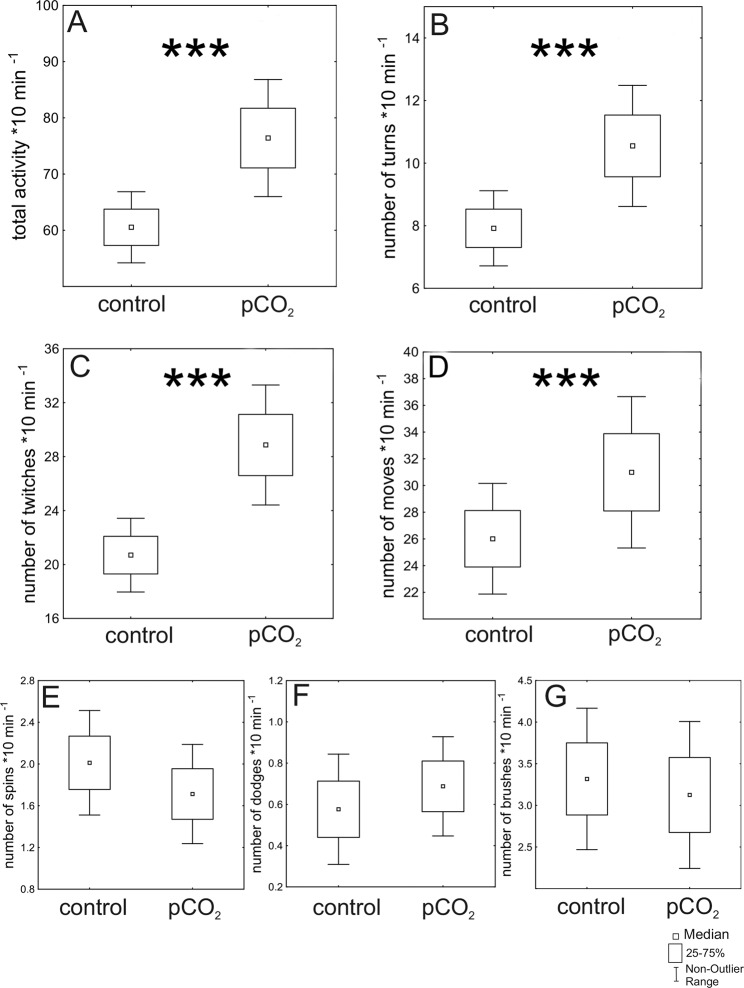
Differences in activity patterns in *Chaoborus* larvae under control and elevated pCO_2_ conditions. (**A**) Total activity is significantly increased in elevated pCO_2_ exposed *Chaoborus* larvae. (**B**) The number of turns, and (**C**) the number twitches, are significantly increased in elevated pCO_2_ exposed larvae. (**D**) the number of moves, is significantly increased under elevated pCO_2_ conditions in comparison to the control. Similarly, (**E**) the number of spins, (**F**) the number of dodges, and (**G**) the number of brushes remain unaffected by increased levels of pCO_2_. Statistics displayed in Table 1.

At elevated pCO_2_, larvae showed significantly increased total activity levels ~1.5 fold from 49.55 ± 21,84 (mean ± StD.) movements in the control condition to 76.40 ± 47.50 (mean ± StD.) movements in the elevated pCO_2_ condition (Table 1, Fig. 1A). The larvae performed significantly more turns increasing ~1.5 fold from 6.92 ± 5.05 (mean ± StD.) turns to 10.55 ± 8,83 (mean ± StD.) turns in the elevated pCO_2_ condition (Table 2 Fig. 1B). Also the number of twitches significantly increased ~1.5 fold from 19.43 ± 11.04 (mean ± StD.) to 28.86 ± 20.30 (mean ± StD.) in the elevated pCO_2_ condition (Table 1, Fig. 1C). The number of moves significantly increased ~1.2 fold from 26.01 ± 19.49 (mean ± StD.) to 30.98 ± 25.87 (mean ± StD.) in the elevated pCO_2_ condition (Table 1, Fig. 1D). Dodges, spins, and brushes did not differ significantly between the treatment and the control (Table 1, Fig. 1D–G).

**Table 1:** Statistical results of activity patterns.

Model	Variable	Estimate	S.E.	z-value	d.f.	P value
Generalized linear mixed model (poisson)	**Total activity**					
	Intercept	4.125	0.044	93.11	162	<0.001***
	Control vs. pCO_2_	0.207	0.019	11.00	162	<0.001***
Generalized linear mixed model (beta)	**Turns**	2.056	0.094	21.794	162	<0.001***
	Intercept	0.2811	0.052	5.452	162	<0.001***
	Control vs. pCO_2_					
Generalized linear mixed model (poisson)	**Twitches**	3.054	0.072	42.454	162	<0.001***
	Intercept	0.296	0.031	9.464	162	<0.001***
	Control vs. pCO_2_					
Generalized linear mixed model (poisson)	**Moves**	3.284	0.036	92.49	162	<0.001***
	Intercept	0.148	0.029	5.09	162	<0.001***
	Control vs. pCO_2_					
Generalized linear mixed model (poisson)	**Spins**	0.693	0.077	9.038	162	<0.001***
	Intercept	−0.1552	0.115	−1.352	162	0.176
	Control vs. pCO_2_					
Generalized linear mixed model (poisson)	**Dodges**					
	Intercept	−0.441	0.145	−3.053	162	<0.01**
	Control vs. pCO_2_	0.061	0.191	0.318	162	0.750
Generalized linear mixed model (poisson)	**Brushes**	1.160	0.154	7.544	162	<0.001***
	Intercept	−0.067	0.087	−0.087	162	0.440
	Control vs. pCO_2_					

Generalized linear mixed model, with time as a random effect component. S.E.: Standard Error, d.f.: degree of freedom of the residuals, *P* values, with significance levels p ≤ 0.05*; p ≤ 0.01**, p ≤ 0.001.

**Table 2:** Statistical results of predation parameters.

Model	Variable	d.f.	Estimate	Std. Error	Z value	*Pr* (>|z|)
Generalized linear model (Poisson)	**Strikes**					
	Intercept	**31**	**3**.**310**	**0**.**0478**	**69**.**266**	**<0**.001***
	Control vs. pCO_2_	**31**	**−0**.**333**	**0**.**074**	**−4**.**501**	**<0**.001***
Beta regression model	**Catches %**					
	Intercept	31	−0.013	0.081	−0.161	0.872
	Control vs. pCO_2_	31	−0.039	0.114	−0.342	0.732
Beta regression model	**Ingestions %**					
	Intercept	**31**	**0**.**995**	**0**.**274**	**3**.**637**	**0**.00276***
	Control vs. pCO_2_	31	−0.199	0.367	−0.516	0.605

Generalized liner model for each parameter (intercept, control and elevated pCO_2_) with specified distributions. D.f.: Degree of freedom of the residuals, S.E.: Standard Error, *Pr* (>|z|): *P* values of z statistics, with significance levels p ≤ 0.05*; p ≤ 0.01**, p ≤ 0.001.

### Predation parameters and predation rate

**Figure 2:**
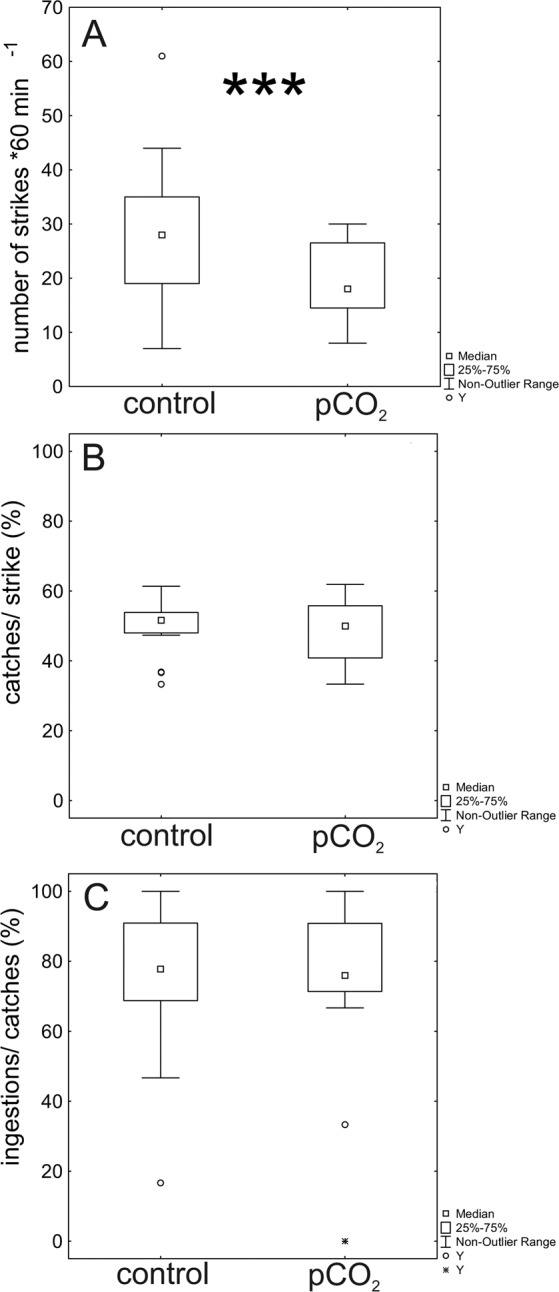
Predation parameters in 4^th^ instar *Chaoborus* larvae under control and elevated pCO_2_ conditions. (**A**) Number of strikes is significantly reduced by elevated pCO_2_. (**B**) Based on the number of performed strikes, the percentage of successful strikes is not significantly different in larvae exposed to elevated pCO_2_ conditions. (**C**) Similarly, the percentage of successful prey ingestion is not affected by elevated pCO_2_. Statistics displayed in Table 2.

We found that pCO_2_ exposed *Chaoborus* made significantly fewer strikes at their prey (Table 2, Fig. 2A). Larvae exposed to elevated levels of pCO_2_ stroke on average ~0.7 fold less in comparison to the larvae of the control conditions. While pCO_2_ exposed larvae performed only 19.63 ± 7.06 (mean ± StD.) strikes at their prey, larvae of the control conditions stroke 27.38 ± 13.47 (mean ± StD.) times. From the performed strikes, we did not observe difference in capture performance, i.e. strikes were similarly effective between both treatments and led to no changes in the amount of captures (Table 2, Fig. 2B). Similarly, ingestions following captures were not significantly different (Table 2, Fig. 2C).

**Figure 3:**
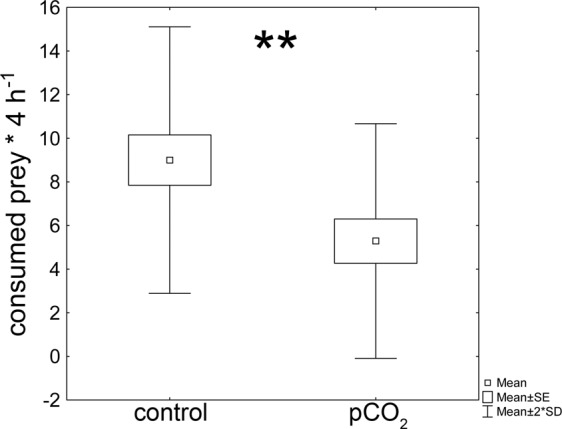
*Chaoborus* predation rate. *Chaoborus* larvae exposed to increased levels of pCO_2_ consumed significantly less *D. pulex*. Statistics displayed in Table 5.

The predation rate was significantly reduced in CO_2_ exposed larvae. Larvae exposed to elevated levels of pCO_2_ consumed ~0.6 less prey; on average only 5.29 ± 2.69 (mean ± StD.) *Daphnia*, while control larvae consumed 9.00 ± 3.06 (mean ± StD.) *Daphnia* (Fig. 3).

**Table 5:** Statistical results of predation rate.

Model	Variable	d.f.	Estimate	Std. Error	Z value	*Pr* (>|z|)
Generalized linear model (Poisson)	**Consumed** ***Daphnia***					
	Intercept	12	1.665	0.1644	3.07	<0.001***
	Control vs. pCO_2_	12	0.5322	0.2071	2.57	=0.01**

Generalized liner model for consumed *Daphnia* (intercept, control and elevated pCO_2_) with specified distribution. D.f.: Degree of freedom of the residuals; S.E.: Standard Error, *Pr* (>|z|): *P* values of z statistics, with significance levels p ≤ 0.05*, p ≤ 0.01**, p ≤ 0.001.

## Discussion

While there is a wealth of research focussing on the effect of ocean acidification on species interactions, only little is known about the effects of elevated pCO_2_ levels in freshwater ecosystems. Up to now there are only a handful of publications investigating pCO_2_ dependent effects in freshwater taxa^24–26^ and community structures^27^. In line with these previous observations, we here observe that *Chaoborus* larvae exposed for 24 h to high levels of pCO_2_ are significantly affected in their behavioural patterns. In these 4^th^ instar larvae we observe behavioural changes in form of increased activity levels accompanied with reduced predatory strikes that result in a reduction of predation rate.

### Increased activity levels

It is already well known, that elevated levels of pCO_2_ alter behavioural patterns in a diversity of marine species (reviewed in^28^). Similarly, some freshwater species showed changes in behaviour^19,29^ while others did not^30^. *Lepomis macrochirus* showed increased swimming velocities^31^, and *Oncorhynchus nerka* was shown to reduce anxiety^19^, while *Gasterosteus aculeatus* showed decreased boldness and curiosity during pCO_2_ elevated conditions^29^. Not only vertebrates are affected by pCO_2_ also other invertebrates, e.g. the freshwater mussel *Lampsilis siliquiidea* shows a reduction of valve movement. Crayfish *Procambarus clarkii* similarly reduced overall activity^20^. Our data contribute to these observations showing that behaviour is also affected in other invertebrates like dipteran larvae. *Chaoborus* larvae exposed to increased pCO_2_ levels increase their overall activity patterns resulting from an increased number of turns and twitches. Directed movements such as forward movements and cleaning patterns (i.e. brushes) or dodges away from conspecifics were not affected. A reason for these increased activity levels may be that larvae try to escape these unfavourable environmental conditions, but this has to be tested in future experiments.

Importantly, our results show that pCO_2_ effects cannot be inferred from other species as increasing and decreasing activity levels are observed. It is quite plausible that such pCO_2_ induced higher activity levels incur energetic costs and higher energy demands.

### Reduced sensory abilities affect predation rate

We find that *Chaoborus* strike less when exposed to elevated pCO_2_ conditions. However, if they strike the probability of prey capture and prey ingestion is not changed. This indicates that not prey handling but prey detection is impaired. *Chaoborus* detect their prey using mechano-sensation^23,32^, which when impaired could explain for the reduced number of strikes. In consequence, we observe that predation rate is significantly reduced, i.e. larvae catch less prey during the same time period. This negatively affects their energy budget, and in combination with the possibly higher energy demand, will have implications for the larvae’s life history parameters, and could affect population growth rates causing changes in community structures. In addition, it is plausible that larvae become more visible for their own predators.

In deed, this may suggest that predation pressure on the prey organism *Daphnia* is reduced. *Daphnia* itself however, are also affected by pCO_2_ as their ability to adequately develop defences is decreased and thereby become more prone to predation^3^. How this will change population dynamics will probably depend on who of the two partners is affected more.

### Mode of action

At present, the precise way how CO_2_ mechanistically affects organisms is still controversial and there are several plausible hypotheses. For example, CO_2_ especially at high concentrations can have narcotic effects on nervous system functionality and could either affect the whole nervous system or only parts that are especially sensitive, thereby disbalancing motor actions and sensory modalities^33,34^.

Another hypothesis focusses on a change in GABA_A_ receptor functioning, where the inhibitory action of GABA is reversed and becomes excitatory^15^. This can result in an increased excitability of the overall nervous system and has the potential to lead to the larvae’s hyperactivity^34^. In an experiment mimicking GABA_A_ receptor malfunctioning with the help of the GABA_A_ receptor antagonist gabazine on *Danio rerio* brains showed an increased spontaneous firing rate which induced epileptic- like neuronal activity^35^. Such neuronal activities stemming from neuronal hyperexcitability could on the behavioural level cause the larvae’s increase in undirected movements. An alternative hypothesis, discusses changes to glycine receptor functioning^34^. Glycine receptors are the dominant inhibitory receptors in many organisms, coupled to an ion channel permeable for chloride ions and carbonate HCO_3_^−^, acting in a similar manner like the GABA_A_ system. It thus, represents an additional explanation of our observations. Which of these hypotheses holds true needs to be subject in future investigations using dedicated strategies e.g. as suggested by^34^.

## Conclusion

Predator - prey interactions are powerful drivers of community dynamics very often regulated via sensory cues passing between predators and prey^28^. As predator and prey, both gather information about the presence of the other, the effects of pCO_2_ increase on predator-prey dynamics will strongly depend on which participant is more compromised. However, the effect of CO_2_ on organismal behaviour is not straightforward but defined by the CO_2_ mode of action which is probably determined by the evolutionary history of the explicit species.

There is strong evidence, that when predator - prey interactions are impeded by anthropogenic stressors such as CO_2_, this may destabilize food-webs and lead to changes in biodiversity.

## Material and Methods

### Animal cultures

*Chaoborus* larvae hatch from eggs deposited in freshwater and pupate into adult midges after processing through four larval stages that are increasing in body size. Due to the gape limitation of their catching basket, they are size selective in their prey choice, and the smaller instars feed on smaller prey items like ciliates, while the larger instars feed on copepods and cladocerans like *D. pulex*^23^. To rule out size selection effects, we choose 4^th^ instar larvae as a representative instar, as these have been well investigated for preying on *D. pulex* in the 2^nd^ juvenile instar^36–38^. This predator-prey system has been well established in the past^36–38^. We anticipate that the results of this instar are well transferable to the other instars. These instars have the same predator capabilities, with the only exception that they prey on smaller items.

All experiments were conducted between September and December of 2018. *Chaoborus obscuripes* larvae of the 4^th^ juvenile instar were caught in the ponds of the Ruhr University’s botanical gardens maximally 5 days prior to the experiments. During this season the ponds have a depth-dependent temperature range of 4 °C to 17 °C. During the summer, when larvae are most active, temperatures can reach up to 25 °C. To acclimate larvae to laboratory conditions, we gradually increased temperature by transferring the larvae from 4 °C via 15 °C to 22 °C in temperature-controlled rooms.

In detail, larvae were isolated from the ponds and twenty individuals were transferred into 1.5 L glass beakers (WECK, Germany) filled with artificial M4 media ((pH 8.0, with a pCO_2_ of ~1,200 µatm, at 4 °C) see Table 3 ^39^), and fed with 50 *D. pulex* juveniles daily. Larvae were first transferred to a cold room at 4 °C ± 1.0 °C for 24 h (16:8 day:night cycle). Subsequently, they were transferred to a room of 15 °C ± 1.0 °C for 48 h, where the medium warmed gradually to carefully acclimate the larvae. They were then transferred to a climatized laboratory set to 22 °C ± 1.0 °C again for gradual acclimation for 48 h. Larvae were not fed 24 h prior to the experiment.

**Table 3:** Composition of a slightly modified M4 artificial *Daphnia* culture medium^39^.

	g/100 mL	concentration	M4 (mL/L)
CaCl_2_ •2H_2_O	29.38	1,000-fold	1.0
MgSO_4_ •7H_2_O	24.66	2,000-fold	0.5
KCl	5.8	10,000-fold	0.1
NaHCO_3_	6.48	1,000-fold	1.0
Na_2_SiO_3_ •9H_2_O	2.5	2,500-fold	0.2
NaNO_3_	0.274	10,000-fold	0.1
KH_2_PO_4_	0.0715	5,000-fold	0.1
K_2_HPO_4_	0.184	10,000-fold	0.1

As prey, we used age- synchronized *D. pulex* (also collected from the botanical gardens, but had been in the department’s animal culture already since 2017). *Daphnia* were also kept in 1 L beakers in M4 at 20 °C ± 0.1 °C (16:8 day:night cycle) in densities of 30 animals per litre. *D. pulex* were fed every 48 h with the green algae *Acutodesmus obliquus*. Beakers were cleaned and water was exchanged on a weekly basis. To match 4^th^ instar *Chaoborus* larvae’s prey spectrum, all experiments were conducted with *D. pulex* that had reached the second juvenile instar^22,38^.

### PCO_2_ conditions and experimental set-up

We set control conditions to a pCO_2_ of ~1,300 μatm (Table 4) with a pH of ~8.0 and elevated pCO_2_ conditions ~12,000 µatm (Table 4) with a pH of ~6.6 as published earlier^3^. These, in comparison to the ocean, high values in the control condition were selected based on the global mean pCO_2_ in freshwater habitats^9^. Similarly, we selected the treatment condition of ~12,000 µatm based on currently observed pCO_2_ maxima of ~10,000 µatm, resulting from the diel and seasonal fluctuations^40^. The elevated pCO_2_ condition was achieved via bubbling and setting the pH to 6.6 prior to the experiments using pH and temperature probes (by Aqua Medic, Germany), documenting temperature levels alongside being stable at ~22 °C. 200 mL of all media were titrated using a Titrino (Methrohm, Switzerland) after the experiments to validate pCO_2_ and temperature conditions. We determined temperature, pH as well as acid and base capacity for pCO_2_ calculation via Phreeqc^3,41^ (see Table 4). The control and the elevated pCO_2_ condition were both tested on the same day but consecutively. To rule out day-time and circadian rhythm dependent effects, we randomized the sequence in which the two treatments were measured. Each experimental trial started between 9 and 10 a.m. for the first condition and between 12 and 1 p.m. for the alternative condition with the exposure of three *Chaoborus* larvae to control and three *Chaoborus* larvae to elevated pCO_2_ conditions for 24 h in custom made water tanks (12.5 cm × 2 cm × 10.5 cm). Tanks were covered airtight by sealing the lid with parafilm to prevent outgassing. All experiments were performed at a constant temperature (see Table 4) in a temperature-controlled room in above mentioned water tanks. On the following day, i.e. 24 h post exposure (i.e. between 9 and 10 a.m. and between 12 and 1 p.m.), the experiments started with the addition of 100 second juvenile instar *D. pulex*. Predator and prey were allowed to acclimate for 10 min. Subsequently, larvae predation parameters were monitored for 1 h. During this monitoring period we additionally recorded 5 film sequences of 10 min using an iPhone 7 (Mac iOS 12.4.2 Apple Inc.) interspaced by 2 min. breaks. For that the iphone was fixed in 13 cm distance from the tank using a tripod (KobraTech, Germany). Iphone camera orientation was positioned in parallel to the frontal plane of the tank. To ensure homogeneous illumination, a diffusor plate (customized translucent PVC plate) was positioned behind the tank illuminated by a 15 W LED lamp (IP 65, LE, Germany). As the larvae are about 1.7 to 2.0 cm in size, this allowed us to record activity patterns and predation parameters in the glass tanks over the experimental period. All experimental trials were replicated 17 times.

**Table 4:** Experimental pCO_2_ and temperature conditions.

Treatment	Valid N	Mean	Minimum	Maximum	Std. Dev.
**Control predation parameters & activity**					
pCO_2_ (µatm)	17	1,391.36	1,000.00	1,995.26	344.78
Temperature (°C)	17	22.36	21.94	24.05	0.554
**CO**_**2**_ **exposed predation parameters & activity**					
pCO_2_ (µatm)	16	10,023.29	9,120.11	11,748.98	718.91
Temperature (°C)	16	22.19	22.40	24.35	0.515
**Control predation rate**					
pCO_2_ (µatm)	7	1,253.808	1,047.13	1,621.81	189.30
Temperature (°C)	7	22.33	20.65	23.7	1.72
**CO**_**2**_ **exposed predation rate**					
pCO_2_	7	10,260.36	9,332.54	11,748.98	819.36
Temperature (°C)	7	22.45	20.70	23.7	1.66

### Analysis of activity patterns

We analysed activity patterns based on recorded videos. Sequences were viewed and analysed using iMovie (Mac OS Mojave Version 10.14.6, Apple inc.). The larvae display distinctive activity patterns, which we categorized into movement categories. A ‘move’ was defined as a forward movement of a larva. A ‘turn’ was defined as a 180° change in orientation, while a ‘spin’ was defined as a full 360° turn around the body axis. A ‘twitch’ was defined as a sudden, undirected convulsive movements. A ‘dodge’ describes the movement, when larvae tried to avoid contact to other larvae. A brush describes a movement in which the larvae clean their tail fan. The category total activity level comprises the sum of all movement categories of the experimental population.

### Predation parameters

During the one hour observation period, we counted all strikes, catches, and ingestions of the larval attacks (according to^22^) and thereby determined the population’s predation parameters. We then calculated the proportion of strikes that led to catches (in %) and the proportion of catches (%) during this one hour.

### Predation rate

To analyse the effects of pCO_2_ on the predation rate of *Chaoborus*, we reared one *Chaoborus* larvae for 24 h in 250 mL M4 either in the control condition or aerated with CO_2_ (Table 4). The experiment started with the addition of 20 second juvenile instar *D. pulex*. After 4 h we counted remaining *Daphnia* and determined the number of consumed animals. We replicated this experiment 7 times.

### Statistics

In total, we performed 17 experimental replicates in the control condition and 17 experimental replicates in the pCO_2_ condition. In the pCO_2_ condition one replicate had to be excluded due to instabilities in pCO_2_ (therefore N_control_ = 17; N_pCO2_ = 16). Activity patterns and predation parameters were calculated as the summated activity of all three larvae and therefore represent the population’s total activity. We recorded activity parameters (i.e. total activity, turns, twitches, dodges, spins, moves and brushes) 5 times (for 10 min) within one hour observation time (N_control_ = 85 and N_pCO2_ = 80). To determine if elevated pCO_2_ has a significant effect on activity patterns we performed generalized linear mixed models (GLMMs) in combination with a poisson distribution for count data, where the different activity parameters were used as response variables, and treatment (control, elevated pCO_2_) was used as fixed effect. As we measured 5 times per 1 h, we included time as a random factor (to reflect a repeated measures design). We fitted the GLMMs using the glmer function implemented in the lme4 package in R; www.raproject.org ^42^).

To analyse count data obtained in the predation parameter ‘strike’ and predation rate, we performed linear mixed models using the glm function and a poisson regression in R. Percent data (i.e. relative catches, and relative ingestions) were analysed using a beta regression using the betareg function in the Betareg package in R according to^43^. As relative ingestion data contained 0 and 1, data was transformed as suggested by^44^ using formula x′ = (x(N − 1) + s)/N (with N = sample size and s = 0.5). All models were validated by visual inspection of the normalised residuals based on the REML fit against fitted values to identify possible violation of homogeneity, according to^45,46^. We tested for overdispersion; a dispersion value of <2 was considered not overdispersed^46^. None of our data was overdispersed.
